# Caspase Dependent and Independent Anti-hematological Malignancy Activity of AMHA1 Attenuated Newcastle Disease Virus

**DOI:** 10.22088/IJMCM.BUMS.8.3.211

**Published:** 2019

**Authors:** Mohammed S. Mohammed, Maha F. Al-Taee, Ahmed Majeed Al-Shammari

**Affiliations:** 1 *Kufa Institute, Al-Furat Al-Awsat University, Iraq.*; 2 *Biotechnology Department, college of Science, University of Baghdad, Baghdad, Iraq.*; 3 *Experimental Therapy Department, Iraqi Center for cancer and medical genetics research, Mustansiriyah University, Baghdad, Iraq.*

**Keywords:** Hematologic neoplasms, oncolytic virotherapy, attenuated NDV

## Abstract

Hematological malignancies remain one of the leading causes of death worldwide despite advances in cancer therapeutics. Newcastle disease virus (NDV) is a member of *Paramyxoviridae *that elicits considerable interest as an anticancer agent because it can replicate up to 10 000 times faster in human cancer cells than in most normal cancer cells. Several NDV strains reportedly induce the cytolysis of cancerous cell lines. The attenuated Iraqi strain (AMHA1) of NDV is a novel oncolytic agent with promising antitumor characteristics, including apoptosis induction. This study aimed to evaluate the ability of the AMHA1 NDV strain to induce apoptotic cell death in hematological tumors through caspase-dependent or independent apoptotic pathways. The cytolytic effects of AMHA1 NDV strains of different multiplicity of infection (MOIs) (20, 15,10, 5, 3, 1, 0.5, and 0.1 )and exposure for all hematological malignancy cell lines (human non-Hodgkin lymphoma SR and human multiple myeloma (COLO 677) and human monocytic leukemia THP1) have been determined through a microtetrazolium (MTT) assay. Propidium iodide and acridine orange (AO/PI) double staining were used to examine the ability of attenuated NDV strain to induce apoptosis in infected cells under a fluorescence microscope and to quantify the percentage of apoptosis induction. Quantitative immunocytochemistry assay was further used to study the caspase-dependent and independent protein expression levels in infected and control cells. Cells treated with NDV strains showed a higher cell-death percentage than untreated cells as quantified by the MTT assay. AO/PI results revealed that NDV exerted a powerful and significant effect on apoptosis induction (P<0.0001) in the human cancer cell lines tested in comparison with control cells. Immunocytochemistry in AMHA1 NDV- infected human hematological cell lines revealed a remarkable increase in the expression of caspase 8, 9 (dependent pathway), apoptosis-inducing factor, and endonuclease G (independent pathway) in comparison with untreated cells. This study demonstrated the role of the Iraqi NDV strain in inducing apoptosis through dependent and independent pathways in cancer cells and thus its high potential as an antitumor agent.

Hematological malignancies are a collection of heterogeneous conditions that originate in bone marrow cells and the lymphatic system. Three groups, namely, leukemia, lymphoma, and plasma-cell neoplasms, are available, and their overall incidence is rising(1), Malignancies are still the leading cause of death despite advances in surgery, chemotherapy, and radiotherapy that is charact-erized by low efficacy and high toxicity for the patient (2). Virotherapy has become a promising option, as the viral replication provides continuous amplification of the injected dose, which continues until stopped by the immune response, and it enters and destroys cancer cells without affecting surrounding normal cells (3, 4). Some viruses, such as Newcastle disease virus (NDV), exhibit the ability to destroy cancer cells. These viruses can be used without any genetic manipulation for increasing selectivity in animal models and human clinical trials (5). NDV can replicate up to 10 000 times better in human cancer cells than in most normal cells (6). NDV causes fatal disease in birds; however, in humans, it is generally not very virulent, causing only mild flu-like symptoms, conjunctivitis, and laryngitis (7). The Avulavirus genus-attenuated AMHA1 Iraqi ND strain belongs to the *Paramyxoviridae* family, and the NDV genome is a single-stranded negative-sense RNA of 15 186 nucleotides (8). The avian NDV is a naturally oncotropic RNA virus (9). Many studies were conducted worldwide using different strains of NDV on different cancer cell lines *in vitro* and *in vivo*. The most important strains were strain 73-T, Lytic strain Italians, NDV strain P701, and NDV strain Ulster. Local NDV strains AF2240 and V4 UPM as oncolytic agents have been studied on breast cancer cell lines (MCF-7 and MDA-231) and brain tumor cell lines (DBTRG.5MG and U87MG) (10-13). NDV replicates rapidly and infects neighboring tumor cells through the release of progeny virions (7). Furthermore, NDV kills tumor cells by modification of the tumor-cell surface and generation of specific cellular immune responses (14, 15). Moreover, NDV can kill tumor cells through induction of apoptosis in the infected cells (16, 17). NDV replication is independent of the host-cell DNA replication that is the target of most chemotherapeutic drugs and radiotherapy and that makes NDV a candidate oncolytic agent to break resistance of tumor cells to the therapies (5).

The present work aims to determine the involvement of a caspase-dependent or independent apoptotic pathway induced by NDV.

## Materials and methods

**Cells and cell culture**

This study was approved by the Baghdad University, College of Science, Biotechnology Department. The human non-Hodgkin (large cell immunoblastic) lymphoma SR cell line (CD20–28) was kindly provided by Dr. SJ Russell, Mayo Clinic, Molecular Medicine Department (Rochester, MN, USA) and cultured in modified Eagle’s media (United States Biological, Salem, MA, USA) with 5% fetal bovine serum (Capricorn Scientific, Ebsdorfergrund, Germany).

The human multiple myelomas (COLO 677) was initially described as being derived from a tumor in the left axillary lymph node of a 39-year old male with small lung cell carcinoma in 1989. However, DNA fingerprinting suggests cross-contamination with cell line RPMI-8226. RPMI-8226 was established from the peripheral blood of a 61-year-old male with multiple myeloma in 1966. The THP1 (human monocytic leukemia) was derived from the peripheral blood of a 1-year-old male with acute monocytic leukemia (18).

HBL-100 normal cell line a continuous cell line (HBL-100) was obtained from primary cultures of cells derived from an early lactation sample of human milk. There was no evidence of a breast lesion in the milk donor. Karyotype analysis showed that all metaphases contained human chromosomes including a large acrocentric marker chromosome. Both desmosomes and cytoplasmic tonofibrils were observed during the early passage. HBL-100 cells exhibited several characteristics of transformation including the ability to form colonies in soft agar, an aneuploid chromosome complement, and continuous growth (19).

The NB4 cell line, a maturation inducible cell line with t(15;17) marker isolated from a human acute promyelocytic leukemia (M3) (20) was obtained from the Department of Experimental Therapy, Iraqi Center for Cancer and Medical Genetic Research (ICCMGR; Baghdad, Iraq). All cells were cultured in Roswell Park Memorial Institute 1640 medium (United States Biological, USA) with 10% fetal calf serum, 100 units/ml penicillin, and 100 μg/ml streptomycin (Capricorn Scientific, Germany) and incubated at 37 °C.

**Viral isolate**

Iraqi isolate of NDV was kindly provided by the Experimental Therapy Department, ICCMGR. The isolate APMV1/Chicken/Iraq-Najaf/ ICCMGR/ 2012 was named AMHA1.

**Propagation of Newcastle disease virus**

A stock of infectious virus was propagated in embryonated chicken eggs, harvested from allantoic fluid, and purified from debris by centrifugation (3000 rpm, 30 min, 4°C). NDV was quantified using a hemagglutination test, in which one hemagglutination unit (HAU) was defined as the smallest virus dose, leading to visible chicken erythrocyte agglutination. Tissue culture infective dose 50% (IC_50_) was determined on VERO cells to calculate virus titer (21).

**Cytotoxicity assay**

The cells were seeded in 96-well plates and were washed with phosphate-buffered saline (PBS) before inoculating with and without the attenuated NDV at different doses (20, 15, 10, 5, 3, 1, 0.5, and 0.1 multiplicity of infection - MOI). After 72 h incubation, the medium was aspirated, and 100 µl of MTT solution (5 mg/ml in PBS, pH 7.2) was added to each well. The plates were incubated for 2 h at 37 °C. After incubation, 50 µl of dimethyl sulfoxide was added to each well, followed by gentle shaking for 45 min to solubilize the formazan dye. The absorbence was determined on a microplate reader at 584 nm wavelength. The assay was performed in triplicate for each of the conditions. The inhibiting rate of cell growth (the percentage of cytotoxicity was calculated as (IG%) =(A−B)/A×100, where A is the mean optical density of untreated wells, and B is the optical density of treated wells (22).

**Quantification of apoptosis using propidium iodide and acridine orange double staining**

AO is an intercalating dye that can permeate live and dead cells. AO will stain all nucleated cells to generate green fluorescence.PI can only enter dead cells with poor membrane integrity. Thus, it will stain all dead nucleated cells to generate red fluorescence. Cells stained with AO and PI fluoresce red because of quenching. As a result, all live nucleated cells fluoresce green, and all dead nucleated cells fluoresce red. 1 µl of AO stock (5 mg/ml) and 1 µl of PI stock (3 mg/ml) was mixed with 1 ml PBS. We added AO/PI to the tested wells after media removal from each well and adding 50 µl of mix solution. After 20 s, we removed the stain from the well and immediately observed under a fluorescent microscope (13). The percentage of apoptotic dead cells was calculated by dividing the number of red cells on the total number of red and green cells (23).

**Quantification of the apoptosis proteins**

The adherent cell line (SR) wascultured on coverslips. The cells were allowed to develop a monolayer. Then, NDV (IC_50_) was exposed for 72 h. Subsequently, the cells were fixed with cold acetone for 2-5 min, then removed using cold acetone, and washed with PBS thrice, and left to dry. After fixation, the slides were incubated in a humidified chamber with 1% H_2_O_2_ for 10 min, washed two to three times with PBS, and incubated with 1.5% blocking reagent for 30-40 min at room temperature. Then, the primary antibody of the CAS-8, CAS-9, apoptosis-inducing factor (AIF), and endonuclease G (EndoG) antibody (Santa Cruz Biotechnology Inc, Dallas, TX, USA) was washed two to three times with PBS for 1-1:30 h. Afterward, the secondary antibody was added and allowed to stand for 2 h. The secondary antibody was then stained by the ImmunoCruz™ mouse ABC staining system (Santa Cruz Biotechnology, USA), washed extensively with PBS, and counterstained with hematoxylin for 30-60 s. The slides were mounted with distyrene, a plasticizer, and xylene (DPX) (supplied by Sigma-Aldrich Co., St Louis, MO, USA), inspected using light microscopy and photographed using a digital camera, the number of positive cells were counted in both treated and control cells manually (3).

To quantify the apoptosis proteins in NB4, THP1 we used a simple indirect enzyme-linked immunosorbent assay (ELISA). Cell lysate of treated and non-treated cells were collected after 72 h of infection and frozen until the analysis was conducted. The concentration of the CAS-8, CAS-9, AIF and endoG was determined by using specific monoclonal antibodies according to the manufacturer’s protocol (Santacruz Biotechnology, USA) by using horseradish peroxidase (HRP) secondary antibody and alkaline phosphatase system. The optical density was determined using microplate reader at 485 nm wavelength. The GraphPad prism software (GraphPad, San Diego, California, USA) was used to analyse the results.

## Results

**Tissue culture infective dose (TCID 50)**

**Fig. 1:**
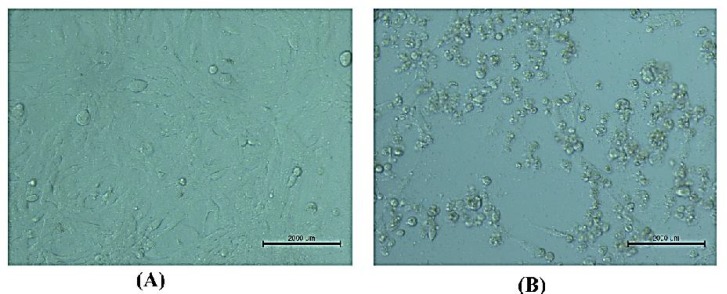
NDV’s cytopathic effect on Vero cells used to titrate viruses (TCID50). **A: Vero cell line not infected with NDV (20×); B: Vero cell line infected with NDV, a cytopathic impact was noted 72 h after exposure**

AMHA1-attenuated NDV strain grew well on the Vero cell line (Figure 1). The cells were used to measure the virus titer in the allantoic fluids after virus propagation in the embryonated chicken eggs.

**Newcastle disease virus has cytolytic effect to hematological malignancies but not to normal cells**

In this study, the cytolytic effects of NDVA MHA1 strain against hematological malignancy cell lines and normal HBL-100 cell line were determined by measuring the cytotoxic dose that kills 50% of the cell population compared with the untreated control for 72 h by using colorimetric cytotoxicity assay (MTT). The assay was repeated three times. The percentage of viable cells was plotted against virus titer (20, 15, 10, 5, 3, 1, 0.5, and 0.1 MOI) and were determined after 72 h of infection. AMHA1-attenuated NDV strain showed cytolytic effect on lymphoma SR cell line, human multiple myeloma (COLO 677), human monocytic leukemia (THP1), and NB4 cell lines in a dose-dependent manner.

**Fig. 2:**
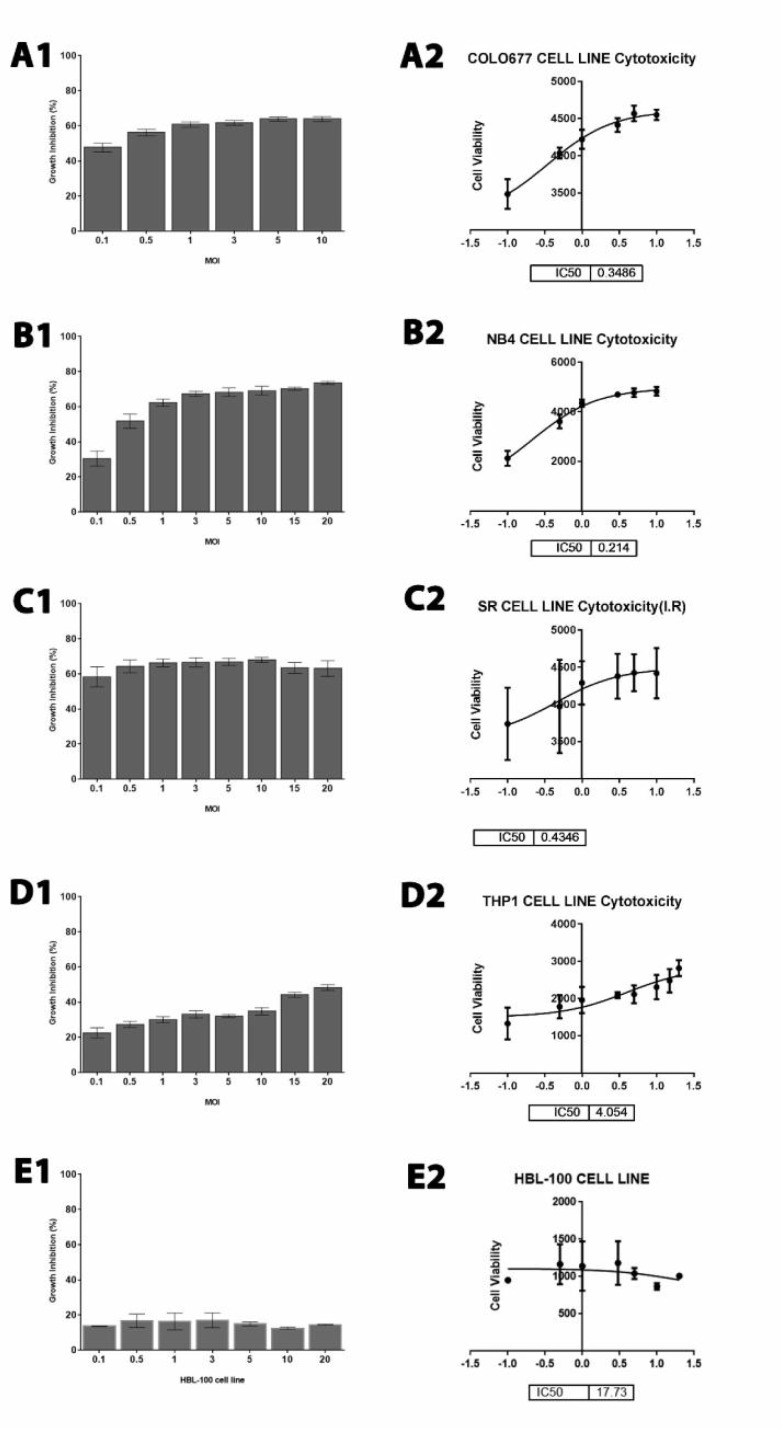
Cytotoxicity effect of NDV on different cell lines. **A1:** **colo677; A2: IC**_50_** of NDV on colo677 cell line (P < 0.0001); B1: cytotoxicity effect of NDV on NB4; B2: IC**_50_** of NDV on NB4 cell line(P < 0.0001); C1: cytotoxicity effect of NDV on SR cell line; C2: IC**_50_** of NDV on SR cell line (P < 0.0001); D1: cytotoxicity effect of NDV on THP1 cell line; D2: IC**_50_** of NDV on THP1 cell line (P < 0.0001); E1: cytotoxicity effect of NDV on HBL-100 (normal cell line); E2: IC**_50_** of NDV on HBL-100 (normal cell line) (P < 0.002), mean± SD.**

The results showed that NDV induced a significant (P <0.0001) cell death and cytotoxicity impact on hematological malignancies. Moreover, we calculated the IC_50_ value for AMHA1 NDV for each hematological malignancy cell line (colo677, NB4, SR, and THP1) and found 0.348, 0.214, 0.434, and 4.04, respectively while the IC_50 _against normal cells was 21.96 (Figure 2). 

**Quantification of apoptosis using propidium iodide and acridine orange double staining**

In this study, a fluorometric cell viability assay by using AO/PI was carried out to detect the morphological changes and the proportions of apoptotic, necrotic, and normal viable cells in the population of SR and NB4 cell lines exposed to attenuated Newcastle disease (NDV) at IC_50_ (0.434 and 0.214 MOI), respectively for 72 h compared with untreated cells. AO will stain all nucleated cells to generate green fluorescence. PI can only enter dead cells with poor membrane integ-rity.Thus, PI will stain all dead nucleated cells to generate red fluorescence. Cells stained with AO and PI fluoresced red because of quenching. As a result, all live nucleated cells fluoresced green, and all dead nucleated cells fluoresced red. Viable cells stained with AO/PI were highly stained with AO and did not incorporate PI and fluoresced green under fluorescence microscopy, whereas nonviable cells fluoresced orange in color. Apoptotic cells were observed in cell lines treated with IC_50_ of AMHA1-attenuated Newcastle disease after 72 h post-inoculation, exhibiting reduced size, integrated membrane, and condensation of chromatin visible as apoptotic cells with bright red/orange areas of condensed chromatin in the nuclei instead of uniform color as observed in necrotic cells. The percentage of apoptotic cells in control SR cells were 3%, but the percentage of apoptotic cells in NDV treatment group was 73.25% which refer to significant apoptotic ability to induce apoptosis by NDV in treated cells in comparison with untreated SR cells. Furthermore, NDV treatment to NB4 cells induced significantly the apoptosis in treated cells which showed 94.54% of total cells undergoing apoptosis in comparison with untreated cells that showed only 5.44% of cells undergoing apoptosis which indicates high ability of NDV to induce apoptosis. 

**Fig. 3:**
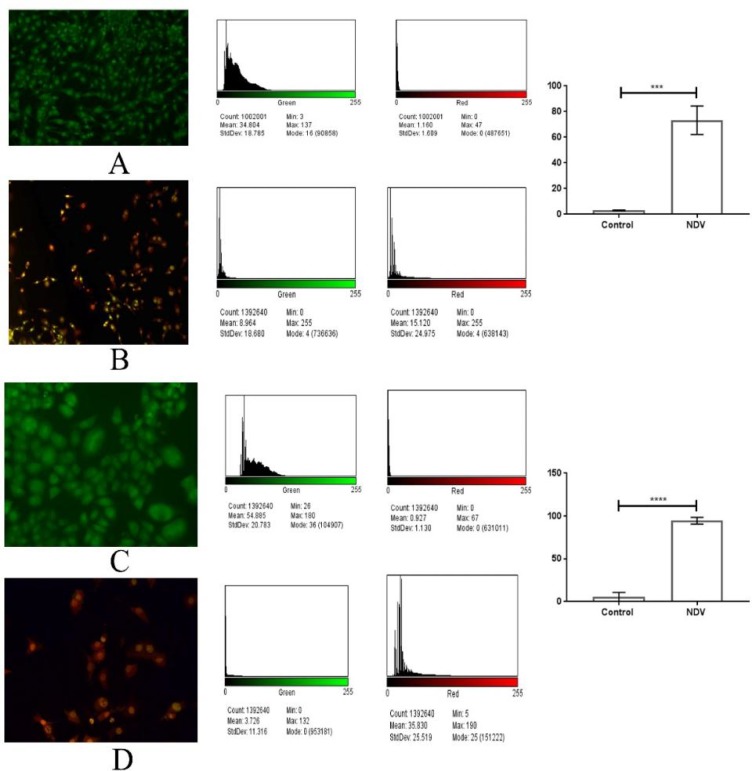
Apoptosis assay by using AO/PI stain. A**: control viable green cells of SR cell line; B: SR cell line infected with NDV showing apoptotic red cells, quantitative image analysis for viable and dead cells showing significant apoptosis induction (P < 0.0004) in the NDV infected cells; C: control viable green cells for NB4 cell line; D: cells infected with NDV showing red NB4 cells, the quantitative image analysis shows that NDV induced apoptosis significantly (P <0.0001) when compared to control not treated cells. Values are presented as mean± SD**

**Quantitative analysis of apoptosis proteins**

**Fig. 4:**
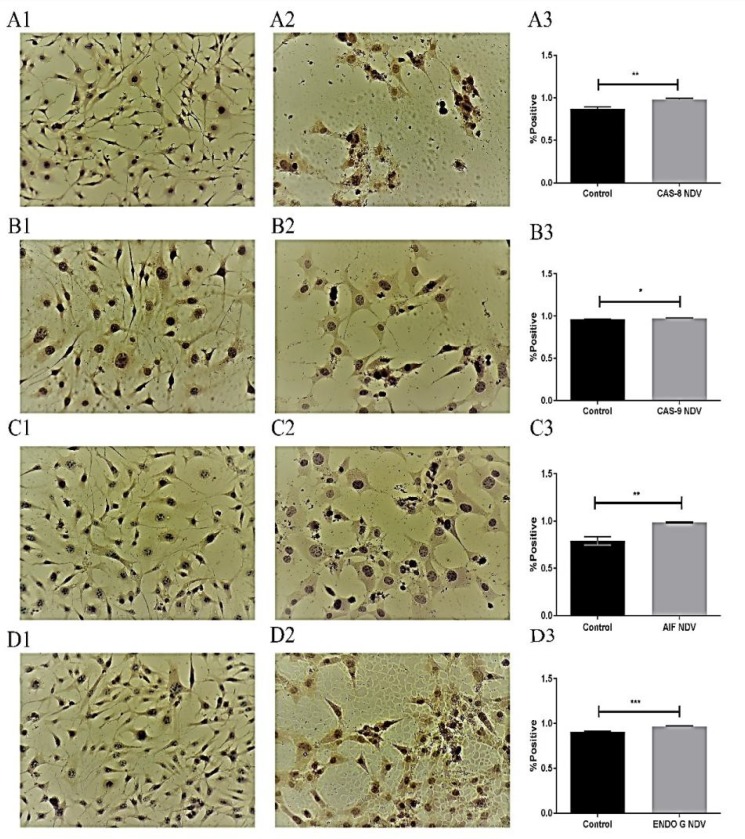
Quantitative immunocytochemistry study of lymphoma SR cell line**. A1: control notuntreated cells; A2: NDV treated cells. Both A1 and A2 were stained with CAS-8 antibody. A3 : analysis showing significantly increased (P < 0.001) caspase-8 expression in treated cells; B1: control untreated and B2: NDV treated cells stained with CAS-9 antibody; B3: analysis revealed significantly increased (P < 0.01) expression in the treated cells; C1:control untreated and C2: NDV treated cells stained with AIF antibody; C3: quantitative image analysis revealed a significant (P < 0.01) induction in the treated cells; D1: control untreated and D2: NDV treated cells stained with Endo G antibody; D3: quantitative image analysis revealed a significant (P < 0.001) induction in the treated cells. (DAB stain). Magnification: 20×. Values are represented as mean ± SD**

Immunocytochemistry results demonstrated that SR cell lines were positive for dependent pathways (CAS-8 and CAS-9) and positive for independent pathways (AIF and EndoG). This test was conducted to confirm that targeted therapy by using attenuated NDV IC_50_ for SR (0.434 MOI) is effective, and the targeted antigen is present. The results demonstrated that the activity of caspase-8 was 88.9% and 112.37% in the control and infected cells, respectively (P< 0.001), and for caspase-9 was 98.5% and 103.45% in the control and infected cells, respectively (P< 0.01) showing a significant increase in both cell lines treated with attenuated NDV. The results also demonstrated that the activity of AIF was 80.22% and 106.38% in the control and infected cells grespectively (P< 0.001), and for Endo G was 94.58% and 124.65% in the control and infected cells, respectively (P< 0.01) (Figure 4). These results were confirmed by using specific monoclonal antibodies for caspase 8, 9 (dependent), and AIF and Endo G proteins for apoptosis. In lymphoma cell line (SR). This test was conducted to identify NDV-inducing apoptosis mechanism *in vitro* after 72 h from infection (antigens in infected cells were stained in brown). Uninfected cells were not stained (nuclei were stained blue by counterstaining). This assay was repeated three times. To quantify the apoptosis proteins in NB4, THP1 we used a simple indirect enzyme-linked immunosorbent assay (ELISA). The results of THP1 cell line demonstrated that the activity of caspase-8 control was 67.88% versus 147.30% in the infected cells (P<0.001), and caspase-9 control was 69.67% versus 143.52% in the infected cells (P<0.001), and the activity of AIF control was 78.99% versus 126.59% in the infected cells (P<0.01), and for Endo G control was 79.02%, versus 126.54 % in the infected cells.

**Fig. 5:**
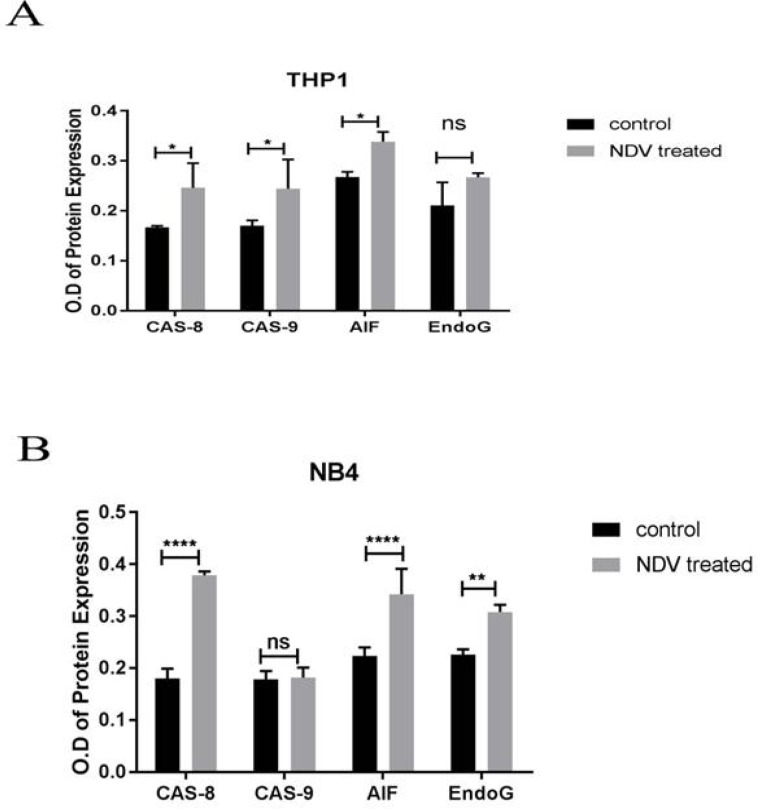
Quantification of the apoptosis proteins in NB4, THP1 using an enzyme-linked immunosorbent assay (ELISA).** A: THP1 cell line treated with NDV was stained with CAS-8 antibody (P = 0.0308094), CAS-9 antibody (P = 0.0461844), AIF antibody (P = 0.00807552), Endo G antibody (P = 0.0713596); B: NB4cell line treated with NDV was stained with CAS-8 antibody (P = 0.000067), CAS-9 antibody (P = 0.829182), AIF antibody (P = 0.001175), Endo G antibody (P = 0.015823). Values are represented as mean ± SD**

The results of NB4 cell line demonstrated that the activity of caspase-8 control was 47.6% versus 186.50% in the infected cells (P<0.000001), and for caspase-9 control was 98.34% verss 101.68% in the infected cells, and the activity of AIF control was 65.2% versus 153.36% in the infected cells (P<0.00001), and for Endo G control was 73.37%, versus 136.28% in the infected cells (P<0.0001) (Figure 5)

## Discussion

This study aimed to investigate the involvement of a caspase-dependent or independent way of apoptosis through infection by oncolytic-attenuated Iraqi NDV strain AMHA1. NDV Iraqi strain is oncolytic and kills tumor cells by intrinsic and extrinsic pathways of apoptosis (24). NDV possesses several unique properties, making it an excellent anti-cancer agent: it is relatively safe, and can act as an adjuvant (25). In current study, MTT cell viability assay showed effective and selective anti-malignant blood tumors in comparison with normal epithelial cells which proves NDV selectivity. NDV has good cell-binding properties, binds specifically to tumor cells, replicates selectively in tumor cells' cytoplasm, is relatively safe, and can act as an adjuvant (13). Schirrmacher et al. (26) used non-virulent lentogenic strain Ulster and found that infection of tumor cells by non-lytic NDV Ulster (30 HU/107 cells) eventually leads to tumor cells' death *in vitro*, and has selectivity in replication in tumor cells (27). Other researchers (28) proved that normal cells resist NDV infection by creating antiviral state that inhibits viral genome amplification through a strong type-I interferon response, leading to persistent production of antiviral proteins. At the same time, they showed that this process in tumor cells is malfunctioning, besides impaired translational control makes cancer cells highly vulnerable to NDV infection.

Morphological and chemical changes of apoptotic cells can be differentiated from any other cell death via many methods. In this study, apoptosis initiated by NDV was confirmed using microscopic examination. AO/PI staining proved that NDV in cell cultures induces cell death via the apoptotic pathway. The number of apoptotic cells revealed that in treated malignant hematological cell lines, the percentage of apoptotic cells proportionally increased with post-inoculation time. Detection using the AO/PI method provides an early indication of the initiation of cellular apoptosis (13).

The oncolytic activity of NDV virus was a consequence of selective induction of apoptosis in tumor cells. Oncolytic activity of a virus is facilitated either by the ability of the virus to replicate better in the tumor compared with non-tumorigenic cells or because of a selective interaction of a viral gene product with a cellular component specific to the tumor cells that induces apoptosis (29).

Quantitative image analysis for the imm-unocytochemistry assay revealed increased expression of caspase-8 and caspase-9 (caspase-dependent pathway) in SR and NB4 cell lines. Caspase-8 was identified as an initiator caspase triggered by death receptors. Thus, the caspase-8 activation suggested that NDV might induce apoptosis through the extrinsic death receptor pathway.

Death receptors are cell-surface cytokine receptors belonging to the tumor necrosis factor receptor superfamily that triggers apoptosis after binding to a group of structurally related ligands or specific antibodies (30). NDV infection of tumor cells produced TNFα and soluble TRAIL in tumor cells and in a virus-specific manner, and resulted in activation of caspase 8. In all tumor cell lines, NDV infection led to the loss of mitochondrial membrane and activation of caspase 9, highlighting the importance of the intrinsic pathway in activation of NDV-mediated apoptosis (31).

The activation of caspase-9 in NDV-treated hematology cell lines can be related to the involvement of mitochondrial pathway. Mitocho-ndria act as important sensors of cellular damage. The result obtained from this study is similar to the study suggesting that apoptosis activation likely proceeds through the mitochondrial pathway and activation of caspase 9, with subsequent activation of caspase 8 (31). However, mitochondrial pathways of apoptosis may not rely on caspases. AIF and Endo G, the two essential death factors in the inter-membrane space of the mitochondria, can be released into the cytoplasm and then translocate into the nucleus under the action of apoptosis-inducing elements. The synergy between these elements causes condensed chromatin and DNA fragmentation and results in caspase-independent apoptosis (32).

In this study, the levels of AIF and Endo G in the cytoplasm of the untreated human hematology cell lines were very low. After treatment with attenuated NDV, the levels of AIF and Endo G increased significantly in the cytoplasm and the nuclei. These data showed that NDV could also induce mitochondrial apoptosis by a caspase-independent pathway in human hematology cell lines. Apoptosis induced by Endo G was shown to be caspase-independent. Endo G revealed to have numerous regulators, including reactive oxygen species (33), apoptosis-inducing factor (34), heat shock protein HSP70 (35). AIF is cleaved and released from mitochondria (36), translocates into the nucleus and triggers caspase-independent apoptosis. Because AIF itself does not have DNase activity (37), AIF was thought to be working together with endonuclease G to promote DNA

degradation (38).

It was also shown previously that the NDV-induced caspase-dependent and independent pathways, AIF, and Endo G could induce caspase-independent apoptotic cell death by directly mediating chromatinolysis (39). This effect allows mitochondrial dysfunction to orchestrate cell death even in the absence of caspase activation (40).

In conclusion, our study suggests that NDV is a very promising anti-hematological malignancy agent that was found to act by inducing apoptosis through caspase-dependent and caspase-indepe-ndent pathways of apoptosis.
